# Individualized Management of Coagulopathy in Patients with End-Stage Liver Disease

**DOI:** 10.3390/diagnostics12123172

**Published:** 2022-12-15

**Authors:** Tina Tomić Mahečić, Robert Baronica, Anna Mrzljak, Ana Boban, Ivona Hanžek, Dora Karmelić, Anđela Babić, Slobodan Mihaljević, Jens Meier

**Affiliations:** 1 Clinic of Anesthesiology, Reanimatology and Intensive Care Medicine, and Pain Treatment, University Hospital Center Zagreb, 10000 Zagreb, Croatia; 2 School of Medicine, University of Zagreb, 10000 Zagreb, Croatia; 3 Liver Transplant Center, Department of Gastroenterology and Hepatology, University Hospital Center Zagreb, 10000 Zagreb, Croatia; 4 Department of Hematology, University Hospital Center Zagreb, 10000 Zagreb, Croatia; 5 Clinic of Anesthesiology and Intensive Care Medicine, Kepler University Clinic, Johannes Kepler University, 4040 Linz, Austria

**Keywords:** ESLD, coagulopathy, cirrhosis, portal hypertension, viscoelastic tests

## Abstract

Over the last decades, individualized approaches and a better understanding of coagulopathy complexity in end-stage liver disease (ESLD) patients has evolved. The risk of both thrombosis and bleeding during minimally invasive interventions or surgery is associated with a worse outcome in this patient population. Despite deranged quantitative and qualitative coagulation laboratory parameters, prophylactic coagulation management is unnecessary for patients who do not bleed. Transfusion of red blood cells (RBCs) and blood products carries independent risks for morbidity and mortality, including modulation of the immune system with increased risk for nosocomial infections. Optimal coagulation management in these complex patients should be based on the analysis of standard coagulation tests (SCTs) and viscoelastic tests (VETs). VETs represent an individualized approach to patients and can provide information about coagulation dynamics in a concise period of time. This narrative review will deliver the pathophysiology of deranged hemostasis in ESLD, explore the difficulties of evaluating the coagulopathies in liver disease patients, and examine the use of VET assays and management of coagulopathy using coagulation factors. Methods: A selective literature search with PubMed as the central database was performed with the following.

## 1. End-Stage Liver Disease (ESLD)

A significant liver disease burden with devastating clinical consequences of end-stage liver disease (ESLD) has positioned cirrhosis as one of the top 10 leading causes of death [1]. From 1990 to 2017, the mortality of cirrhosis increased by 47.15%, mainly due to hepatitis C virus (HCV), alcohol consumption, and non-alcoholic steatohepatitis (NASH), while hepatitis B virus (HBV) and other causes showed a decreasing trend [2].

ESDL is accompanied by portal hypertension (PH), the major reason for many complications associated with ESLD. As a consequence, treatment of PH is a cornerstone in any ESDL treatment algorithm. Additionally, decompensation rates and patients’ survival are affected by the underlying etiologies [3]. However, regardless of the etiology, current treatment options for ESLD are limited [4]. Drugs that decrease hyperdynamic circulation (non-selective beta blockers) and diuretics for the treatment of hypervolemia are currently the mainstream treatment [5]. In addition to PH, dysregulation of the coagulation cascade is common in ESLD and simultaneously affects procoagulant and anticoagulant mechanisms. The predominance of either of them can lead to prolonged bleeding or thrombotic events [6].

Individualized approaches and a better understanding of coagulopathy complexity in ESLD patients evolved over the last decades [7], allowing for a better treatment of patients during invasive interventions and surgical procedures [8]. Liver transplantation (LT) is the definitive treatment for ESLD; however, sources are scarce. Therapeutic plasma exchange (TPE) is a possible bridge to LT by removing accumulated toxins from patient plasma and by transitorily improving the coagulation profile and liver function tests in ESLD patients [9].

As the morbidity of ESLD rises, clinical experience with ESLD consequences is expanding. This comprehensive review addresses one of these consequences—coagulopathy—examining the pathophysiology of deranged hemostasis in ESLD, difficulties of evaluating patients with chronic liver disease, the use of viscoelastic tests (VET) analysis, and management of coagulopathy.

## 2. Pathophysiology of Deranged Hemostasis in End-Stage Liver Disease

Normal hemostasis is a complex interaction of platelets, endothelial surface, and plasmatic coagulation factors with inhibition pathways [10,11].

Under physiological conditions, the endothelium as well as platelets are negatively charged. As a consequence, they mutually repel each other until the blood vessel wall is damaged. The disintegration of the endothelial barrier results in exposure of positively charged proteins, mainly von Willebrand factor (vWF), which is expressed on subendothelial cells. vWF activates platelets GPIa/IX receptor. Activation results in expression of GPIIb/IIIa on the platelet membrane, which is the target protein for fibrinogen binding, the most important step for the formation of the initial clot. Up to this point, this process is called “primary hemostasis” [12]. In ESDL patients this part of hemostasis is preserved. The result of this cascade is the release of tissue factor (TF) from the endothelial cells and macrophages, which is the initiator of the “secondary hemostasis”, which comprehends the plasmatic coagulation factor cascade [12].

In patients with ESLD, there is a decrease in general protein synthesis.

Coagulation factors dependent on vitamin K, such as FII, FVII, FIX, and FX, are significantly decreased, as are the other coagulation factors produced by the liver, such as FV, FXI, FXII, FXIII, fibrinogen, and anticoagulant factors, such as proteins C and S and antithrombin. To sum up, the activity of both pro- and anticoagulant factors is decreased, resulting in a new rebalance in hemostasis [11,13].

On the contrary, liver-independent coagulation factors produced from the endothelium, such as FVIII and vWF, are increased [14,15]. vWF binds FVIII and prevents its degradation with plasmatic proteases, which leads to increased levels of FVIII and an increase in the generation of thrombin with the consequence of an increased risk of thromboembolic incidents.

ADAMTS13 (a disintegrin and metalloproteinase with a thrombospondin), a metalloprotease enzyme (synthesized in the liver) that breaks down vWF, is decreased in ESLD patients. The low-level activity of ADAMTS13 in plasma and increased release of vWF from endothelial cells is the reason why ESLD patients can compensate for low platelet levels [15].

With the progress of portal hypertension, thrombocytopenia worsens due to increased platelet sequestration in the spleen. Additionally, platelets have shortened life spans due to antibody-mediated destruction [16], relative bone marrow insufficiency, and decreased thrombopoietin secretion in a failing liver. However, thrombocytopenia below 50,000/μL is seen in less than 1% of patients. Systemic inflammation can further reduce ADAMTS13 activity, and despite severe thrombocytopenia, patients can still be paradoxically prothrombotic. For additional rebalance, ESLD patients have increased nitric oxide production and increased levels of prostacyclin. Those mediators are released from the endothelium of ESDL patients, preventing platelet activation [17].

Endogenous thrombin potential (ETP) in vitro is decreased in cirrhotic patients compared with healthy volunteers and their international normalization ratio (INR) value is higher [18].

Reduced levels of procoagulant factors typically found in ESLD patients influence thrombin generation (TG) measured without thrombomodulin (TM). However, by adding TM, (membrane protein, produced by endothelial cells, not by the liver that activates protein C in vivo) in the test, as a result, patients generated as much thrombin as controls. Two different situations can be distinguished: TG can be tested with or without activating the protein C pathway. In the first case, a hypercoagulable state can be diagnosed, whereas in the second case the patient is prone to a reduction in the coagulation potential [18]. Especially if the platelet count is above 75,000/μL, primary hemostasis and thrombin production do not appear to be decreased in ESLD patients [19].

At the end of the amplification phase of the coagulation process, the thrombin burst induces the formation of the fibrin net and its cross-link with F XIII to enhance the clot strength; in ESLD patients, the fibrin clot is not stable [20]. This weak clot simultaneously increases the risk of bleeding and paradoxical embolic incidents if the clot disintegrates and is released into circulation.

**Figure 1:**
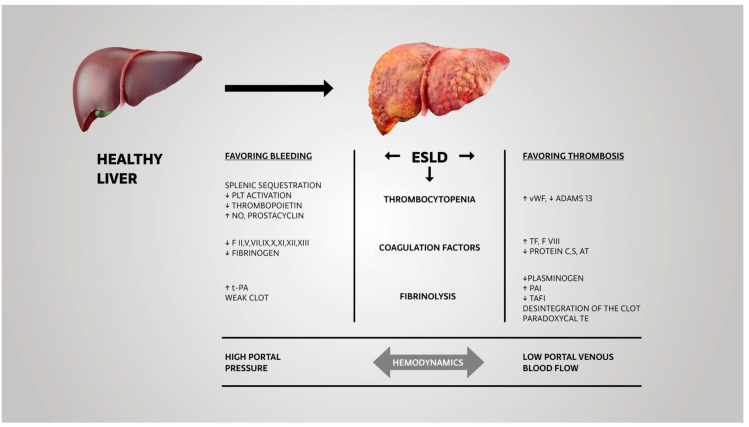
Factors contributing to deranged hemostasis in end-stage liver disease (ESLD). Abbreviations: ESLD—end-stage liver disease, PLT—platelets, NO—nitric oxide, F—factor, t-PA—tissue plasminogen activator, vWF—Von Willebrand factor, AT—antithrombin, PAI—plasminogen activator inhibitor, TAFI—thrombin-activatable fibrinolysis inhibitor, TE—thromboembolism.

As the last step in the coagulation process, the dissolution of a clot with fibrinolysis is also deranged in ESLD patients. Thrombin-activatable fibrinolysis inhibitor (TAFI) levels are decreased due to impaired synthesis in ESLD patients, depending on the severity of the disease [17]. ESLD patients have reduced levels of plasminogen and increased levels of plasminogen activator inhibitor (PAI). Therefore, they are prone to hypofibrinolysis rather than lack of hyperfibrinolysis, which aggravates the risk for thrombosis [17]. (Figure 1)

During the last decades, ESLD patients have been found to have an increased risk for thrombosis [21]. Ben-Ari et al., found that patients with cholestatic liver disease were hypercoagulable compared to healthy volunteers [22]. Data from the Denmark registry databank of patients with thrombosis and pulmonary embolism indicate a higher relative risk for hypercoagulable states in ESLD patients than in patients without liver diseases [21]. In particular, in ESLD patients, prolonged prothrombin time (PT)/INR and activated partial thrombin time (aPTT), in combination with a diminished activity of factor V, is an indicator for an increase of the thrombotic risk but not the bleeding risk [23].

This situation is even worse for patients with hepatocellular carcinoma (HCC): ESLD has an increased prothrombotic profile in this setting. It has been demonstrated in 50 patients with a median HCC volume of 9 cm^3^ (range: 5–16 cm^3^) that there is an increase in prothrombotic hemostatic profile due to increased thrombomodulin and reduced activation of fibrinolysis compared to controls without HCC. [24].

For the treatment and prevention of venous thromboembolism (VTE) in ESDL patients, all types of anticoagulants could be used, including heparin, vitamin K antagonists (VKAs) such as warfarin, and direct oral anticoagulants (DOACs). Nevertheless, patients with ESLD may have resistance to heparin due to antithrombin deficiency. Anticoagulants are most effective in the prevention and management of portal vein thrombosis (PVT) [25]. Treatment of PVT in ESLD requires careful assessment of the bleeding risk and individualized decisions for or against anticoagulation [26].

Today, anticoagulation is strongly recommended to improve post-transplant outcomes for patients with PVT, who are candidates for LT surgery [27]. However, prophylaxis in these patients is challenging in the case of bleeding complications. In the setting of hemostatic dysfunction secondary to ESLD, increased fibrinolysis, even without antithrombotic medications, potentially augments the risk of potentially lethal spontaneous subdural hematoma [28]. Considering the risk of bleeding, antithrombotic drugs should be interrupted before invasive procedures or planned interventions.

ESLD patients are complex because of the high risk of bleeding and simultaneous thrombosis in different clinical scenarios [29]. Still, bleeding is mainly related to portal hypertension and less to coagulopathies [19].

The predilection sites for portal-hypertension-induced acute bleeding in ESLD patients are mainly „gastrointestinal varices“ typically found in the esophagus, stomach, or rectum. Variceal bleeding is very common in this group of patients and is one of the main drivers of ESLD-associated allogeneic blood transfusions [30]. Recently it has been demonstrated that allogeneic RBC transfusions are associated with higher mortality in patients with variceal bleeding compared to other bleeding patients [31]. In addition to pharmacological interventions that influence portal perfusion pressure, such as terlipressin, somatostatin, or octreotide, coagulation factors have a major role in the treatment of variceal bleeding. A liberal application of RBCs, FFPs, or platelets increases intravascular volume and thus the tendency for pressure-induced bleeding in this situation [32].

In particular, it is difficult to predict whether an ESLD patient is more likely to be pro- or anticoagulant, since both parts of the coagulation system are affected, which makes treatment of these patients a real challenge [33].

## 3. Difficulties with Evaluating the Coagulopathies in ESLD Patients

Low levels of platelets and deranged standard laboratory tests (SLTs) have traditionally been warning signs for a high risk of bleeding in all patients, especially in those with ESLD.

There is a growing body of evidence that indicates how SLTs do not truly reflect an objective risk for bleeding, especially PT-prothrombin time and aPTT-activated partial thrombin time [34] due to the time course of clot formation. These parameters are only descriptive for the first 5–10% of fibrin formation. However, clot strength and stability and the interaction of all elements of the coagulation system with the vessel wall cannot be analyzed with these parameters [35,36]. SLTs are insensitive to coagulation inhibitors which are also decreased in liver dysfunction where there is rebalanced hemostasis with a combination of higher vWF and FVIII and lowered physiological anticoagulants [37,38]. The PT and APTT are unlikely to predict the hemostatic status of ESLD patients since they are only sensitive to procoagulant proteins [20,39,40,41], and anticoagulant activity is not measured [16].

Over the years, it has become more evident that these laboratory values cannot serve as a reliable indicator of bleeding risk in these patients since stable cirrhotic patients have a rebalanced hemostatic system and preserved thrombin generation in vivo [36,40,41,42,43].

Even though ESLD patients are traditionally considered at high risk of bleeding during invasive procedures, the association and correlation with conventional coagulation tests are still controversial [44]. Napolitano et al. analyzed data on platelet count and INR values in an extensive series of cirrhotic patients who underwent invasive procedures and found that post-procedural bleeding was rare and could not be predicted by platelet counts or abnormal INR values [16]. A similar conclusion was made by Shah et al., who demonstrated no significant bleeding after invasive procedures and nonsignificant periprocedural correction of abnormal coagulation parameters with plasma or platelets [45].

Aside from insufficient accuracy in using INR and platelet count to predict bleeding, Janko et al. also showed that restoring these parameters with blood components failed [46]. This is consistent with findings in other papers that transfusion provides only partial and transient correction of the laboratory derangements regardless of the number of units transfused [47].

The meta-analysis by Haas et al., which evaluated the importance of SLTs, found no strong evidence that SLTs provide either security or are conclusive for predicting bleeding during invasive procedures [48]. In patients undergoing LT, neither PT nor INR can predict the risk of bleeding, nor does prolonged INR exclude patients’ hypercoagulability [49]. These findings are also part of the British Society for Haematology guidelines (Grade 1b) [50]. In addition, Lawson et al. showed that INR is not a reliable tool for predicting massive transfusion during LT, regardless of the small number of study participants [51]. Not to be disregarded, INR results may differ between laboratories by up to 0.7 depending on the available reagents [47].

Mild to moderate thrombocytopenia is usually present in patients with ESLD. Some studies associate thrombocytopenia with the risk of bleeding during invasive procedures, demonstrating the delicacy of balancing hemostasis in different conditions [36,39]. A retrospective study on over 800 patients showed that, even though platelet count lower than 50 × 10^9^/L was associated with major bleedings after invasive procedures, there was no significant relationship with INR of 1.5 and higher [44].

Therefore, standard coagulation tests and platelet counts are inadequate for estimating the hemostatic status of ESLD patients and deliver insufficient data for predicting a balance between bleeding and thrombotic complications [20]. Furthermore, this state of new rebalance can be deranged by the onset of sepsis or any acute insult [52].

This situation is complicated by the fact that patients with ESLD can have thrombosis and bleeding at the same time. Whereas there is thrombosis at one site (e.g., portal vein or mesenteric thrombosis), there can be concomitant severe bleeding at another site (e.g., variceal bleeding).

## 4. Use of Viscoelastic Tests Assays

Two main viscoelastic tests (VETs) are currently widely used; thromboelastography (TEG), developed in 1948 [53], and/or thromboelastometry (TEM/ROTEM), the technical modification of TEG.

Both VET methods usually provide a visual graph of the clot formation process, including initiation of coagulation, clot strength, and clot lysis.

VETs have the advantage over SLTs because the analysis is based on clot assessment in whole blood, considering the balance and interactions between the pro- and anticoagulants and the importance of platelets [54]. A cohort study of ESLD patients showed that clot initiation and propagation are preserved, whereas maximal clot strength is impaired [55]. In addition, this study showed that VETs do not correlate well with traditional measures of hemostasis in ESLD patients and that VETs are not associated with the outcomes [55]. However, VETs results can be associated with disease severity [56].

There is an emerging amount of evidence demonstrating the superiority of VETs for assessing bleeding risks with the aim to avoid transfusions. The safety profile of these VETs is similar in ESLD patients from minimally invasive to invasive procedures and LT surgery. In a prospective trial from Bedreli et al., the authors proved that, compared with SLT-guided hemostasis treatment in minimally invasive procedures, ROTEM-guided coagulation management can reduce the use of coagulation factors without increasing bleeding complications [57]. Another randomized control trial (RCT) by De Pietri et al. included 60 ESLD patients who underwent different interventions or surgical procedures [58]. They were randomized into two groups, VETs versus SLTs. Trigger values for intervention were INR and platelet number in the SLTs group and in the VETs group reaction time and maximal amplitude. All patients in the SLTs group received at least one of the blood components (red blood cell (RBC), FFP, and platelets), and in the VETs group, only 16% of patients were transfused. Clinically significant bleeding episodes were more frequently observed in the SLT group but without statistical significance.

Although in the last several decades many publications on the successful use of VETs in LT surgery have been published, physicians are nowadays still reserved about using VETs for the perioperative optimization of hemostasis. Transplantation surgery itself presents additional hemostatic disbalances, including hyperfibrinolysis [59]. As early proof in the study by Kang et al., it was shown that TEG was associated with a 33% reduction in blood and fluid infusion volume, with maintained blood coagulability [60]. Additional proof favoring the use of VETs in ESLD patients during LT is found in work by Wang et al. [61]. Despite a low number of patients (28), the authors found that in patients monitored via TEG, those who were randomized in the VETs versus SLTs group needed significantly less FFP (mean: 12.8 vs. 21.5 units). Furthermore, it is important to mention that the 3-year survival in both groups did not differ.

A prospective trial with more than 200 LT patients divided participating patients into SLTs or ROTEM-guided hemostatic management [62]. In the ROTEM group, the number of transplanted patients not requiring any blood products significantly increased from 5 to 24% (*p* < 0.001). Furthermore, the patients treated with a ROTEM based algorithm were less prone to reoperation, acute kidney failure, or hemodynamic instability.

Additionally, Al Moosawi et al. suggested that using ROTEM in LT surgery may prevent unnecessary transfusions of FFP for isolated elevated INRs, proving an advantage for coagulation assessment in ESLD patients compared to INR [63]. In a retrospective observational study by Gaspari et al., a preliminary analysis showed that patients in the TEG group, compared with the SLTs group, received fewer RBCs and blood products. After propensity score matching, investigators found no difference between the groups, except in a subgroup of 39 patients with MELD ≥25, where viscoelastic testing significantly decreased the number of FFPs as well as the number of total blood products used [64].

The amplitude of the clot strength after 5–10 min has been demonstrated to be positively correlated with the maximum clot firmness [65,66,67], which, as a consequence, can be used as a fast prediction [68]. This is of major importance because time is the enemy of bleeding patients.

Avoiding the tendency of clinicians to use ROTEM as a last resort in patients with severe ESLD who require more tests and blood transfusions as a part of their management [69] raises the need for implementing ROTEM-based coagulation management algorithms. Timely and rational use of coagulation based ROTEM algorithms results in an overall reduction of FFP and RBC transfusions without the concomitant risk of complications [20].

The working group on “Intraoperative Transfusion Management, Antifibrinolytic Therapy, Coagulation Monitoring”, and the working group for the “Impact on Short-Term Outcomes after Liver Transplantations” of the Enhanced Recovery after Liver Transplantation Society recommends the usage of VETs, even if their implementation results in increased factor use. The experts state that VETs in combination with a transfusion protocols result in less allogenic blood transfusions compared to conventional coagulation tests [70] and as such are highly cost-effective [71].

## 5. Timely Management of Coagulopathy by Using Coagulation Factors

Transfusion of blood and blood products carries independent risks for morbidity and mortality, including modulation of the immune system with increased risk for nosocomial infections [72].

The old-fashioned approach for managing coagulopathy was proactive transfusion based on SLTs values to prevent potential bleeding complications. However, abnormal coagulation parameters do not necessarily assume a higher risk for bleeding in patients with ESLD, and the decision to correct them by transfusion should consider the clinical context [73].

It has been demonstrated in ESLD patients that mortality significantly increases (43% with transfusion vs. 5% without transfusion) if intraoperative blood transfusions are given [74]. However, the exact physiological reasons for this phenomenon remain unclear. It is probably influenced by the combination of surgical stress and transfusion-associated immunomodulation [72] and also cirrhosis-related factors, such as coagulopathy, collateral vessels, and increased portal pressure, might be responsible.

ESLD patients undergoing non-hepatic surgery require intraoperative blood transfusion more frequently (almost doubled) than those without liver disease [75]. Intraoperative blood transfusions represent an independent risk factor for developing cirrhosis-related complications.

The severity of these complications and their impact on outcomes are associated with prolonged PT, INR, and intraoperative transfusion of FFP [74,75,76].

The main challenge during the correction of coagulopathy in ESLD patients is not only to prevent and control bleeding but, at the same time, to also prevent potential thrombosis. The procoagulant state in ESLD patients, due to bacterial translocation and systemic inflammation, can be seen on ROTEM results and compared with inflammation markers such as C-reactive protein (CRP), procalcitonin, and leukocyte count [77]. Additionally, acquired bacterial infections, common in decompensated ESLD patients, increase the risk of bleeding during invasive procedures. A prospective study on ESLD patients with and without bacterial infections showed that bacterial infections are associated with decreased procoagulant FVII and FXII and significantly lower values of all-natural anticoagulants, which again aggravate the risk for bleeding and thrombosis in the same patient [78].

Summing up, it can be stated that taking the studies published so far into consideration, a restrictive coagulation management in ESLD patients is warranted as long as patients are not actively bleeding [79].

### 5.1. Platelets

Recommendations for platelet transfusions are primarily based on expert opinions.

In nonhepatic, general abdominal surgery procedures in ESLD patients, without any direct liver trauma with concomitant postoperative liver failure, platelet counts of less than 100 × 10^3^–150 × 10^3^/μL were associated with an increase of mortality [80,81].

In hepatic surgery procedures, successful hepatectomy has been reported despite thrombocytopenia [82,83]. Cucchetti and colleagues demonstrated, in a propensity-score-matched analysis of patients with and without portal hypertension, that hepatectomy for HCC can be performed safely even in patients with severe thrombocytopenia [82]. In this study the degree of the hepatic reserve was more important for the postoperative outcome than the platelet number.

As an alternative to platelet transfusion in acute thrombocytopenia, the administration of thrombopoietin (TPO) receptor agonists could also be an option. TPO is a cytokine with action mediated by a specific receptor on megakaryocytes resulting in an increase of the number of platelets [84]. TPO receptor agonists have the same effect and are used successfully in the therapy of acute thrombocytopenia [85]. The theoretical advantage compared to platelet transfusion is a lower transfusion-associated risk caused by a lower immunological burden. However, depending on the product used, the effectiveness may be highly variable [86]. Larger studies that could describe the side effect profile of TPO-R agonists with ESLD patients are currently lacking.

### 5.2. Coagulation Factors

Nowadays the most recent literature demonstrated that hepatic coagulopathy should be preferably treated with coagulation factors, and not FFPs due to the positive benefit/risk ratio (efficacy vs. infection or volume overload).

Transfusion of FFP, routine in the past for correction of hemostasis disorders in ESLD patients, is additional volume overload and can aggravate portal venous pressure. Increased portal venous pressure might significantly increase bleeding [87].

In the multicenter prospective cohort study on 1299 patients who received FFP due to liver-associated coagulopathy, FFP was identified as an independent 28-day mortality risk factor [88]. Patients with ESLD often have chronic coagulopathy, which does not improve with FFP transfusion alone.

There are alternatives for FFP in treating inadequate thrombin generation, such as FC, cryoprecipitate, or PCC.

Fibrinogen and platelets are the main contributors to clot firmness [89]. Fibrinogen is not only an important coagulation factor for building a stable clot but also has a favorable safety profile. Fibrinogen concentrate allows significant clot strengthening after graft reperfusion in LT surgery [90]. Due to the absence of vWF, FVIII, and FXIII, its administration is theoretically less prothrombotic compared to cryoprecipitate.

However, the increased formation of poor-quality clots and fibrinolysis leads to a clinical condition similar to disseminated intravascular coagulation (DIC), labeled in ESLD patients as “accelerated intravascular coagulation and fibrinolysis” (AICF), with elevated D-dimer level, low fibrinogen, elevated PT/APTT, and progressive thrombocytopenia. AICF correlates with mortality and severity of liver disease. The patients usually bleed from mucosae and intravascular lines. Predictors of bleeding include fibrinogen level < 0.6 g/L, platelet count < 30 × 109/L, and APTT > 100 s. In a retrospective, multicenter study, 4F-PCC, Prothrombinex^®^-VF, an Australian-produced, four-factor prothrombin complex concentrate that contains factors II, IX, X, and low levels of factor VII, was found to be contraindicated in DIC and also should be used with caution in ESLD [91]. Without meaningful improvement in coagulopathy, ESLD patients in this study were prone to develop AICF/DIC following Prothrombinex^®^-VF.

Due to their composition, four-factor prothrombin complex concentrates (4F PCC) are considered to be effective and safe in the treatment of hepatic coagulopathy [20]. It has been demonstrated, using 4F PCC preparations which contain vitamin K-dependent procoagulants (factor II, VII, IX, and X), as well as vitamin K-dependent anticoagulants (proteins C and S), is safe regarding thrombotic complications [92].

Initially, antithrombin (AT) was used prophylactically to prevent the possibility of overtreatment with PCCs and possible thrombotic complications. Today, with VETs controls, AT usage can be unnecessary.

Additionally, thrombotic events were not detected in antivitamin K agent (AVK) reversal with PCC in ESLD patients awaiting LT. However, the effect of reversion with PCC is questionable since in one study there were no differences between patients who received PCC and those who did not in intraoperative blood loss, RBCs, fibrinogen, platelet transfusion, or postoperative bleeding [93].

### 5.3. Antifibrinolytics

Outside LT surgery, little data exist that evaluate the use of antifibrinolytic agents in hepatic coagulopathy, even for tranexamic acid (TXA), which is widely used in many clinical situations.

ESLD patients in LT surgery frequently develop hyperfibrinolysis (range 5–84%), mainly during the reperfusion phase of the transplanted liver [94,95]. With today’s knowledge, fibrinolysis can be self-limiting and should only be treated when it occurs parallel to excessive bleeding.

The “Enhanced Recovery after Liver Transplantation” expert panel does not recommend a specific blood transfusion practice in LT surgery. The panel emphasizes that packed red blood cell transfusion (PRBC) and platelet transfusions are associated with higher mortality. However, the use of PCC and FC is not associated with increased thrombotic events in LT. No routine use of antifibrinolytics in LT is recommended [70]. In addition, basic conditions, hypothermia, hypocalcemia, and acidosis worsen coagulopathy [96]. During LT surgery, metabolic acidosis is independently associated with decreased fibrinogen levels and increased intraoperative transfusion requirements [97].

**Figure 2:**
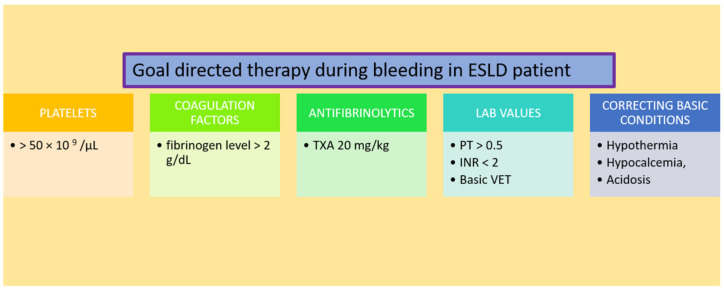
Target values for goal directed therapy of bleeding in ESLD patient. Abbreviations: TXA—tranexamic acid, PT—prothrombin time, INR—international normalized ratio, VET—viscoelastic tests.

In ESLD patients with active surgical bleeding, the authors’ transfusion practice is presented in Figure 2.

Finally, important issues during bleeding in the operating room are adequate communication skills and safe, confident, and patient-orientated teamwork [98,99].

## 6. Conclusions

Hemostasis in ESLD is demanding for clinicians, knowing that despite being newly rebalanced, individual patients are at high risk for thrombotic incidents and inappropriate bleeding during invasive procedures. In the absence of bleeding, prophylactic coagulation management is unnecessary or even harmful in ESLD patients due to prothrombotic risks.

Coagulation management should be based on VETs analysis. That enables reactions on time, is intuitive, and allows us to recognize all coagulation dynamics.

Based on the recent data, ESLD coagulopathy is a complex issue, not easily recognized by in vitro SLTs, and routine transfusion of FFP is not the answer. Coagulopathy of ESLD patients is managed with coagulation factors rather than FFP alone due to high efficacy and an advantageous safety profile. More clinical research is needed for targeted transfusion protocols and pharmacologic prevention of fibrinolysis to manage complex ESLD patients with liver-associated coagulopathy.

## Data Availability

Not applicable.
